# Etiology-related differences in hepatic recompensation in decompensated cirrhosis under Baveno VII criteria: a systematic review and meta-analysis

**DOI:** 10.1186/s12876-026-05087-8

**Published:** 2026-07-08

**Authors:** Jia-Ying Wang, Meng-Xiao Zhang, Shao-Hui Su, Shan-Hong Tang

**Affiliations:** 1 Department of Gastroenterology, Chinese People’s Liberation Army Western Theater General Hospital, Chengdu, Sichuan Province 610083 China; 2 College of Medicine and Life Sciences, Chengdu University of Traditional Chinese Medicine, Chengdu, Sichuan Province 610072 China

**Keywords:** Cirrhosis, Recompensation, Baveno VII criteria, Etiology

## Abstract

**Background:**

Decompensated cirrhosis has different causes. These causes differ in how they damage the liver, how difficult they are to treat, and how well they can be controlled. This may lead to different prevalences of recompensation. However, existing studies have failed to elucidate the heterogeneity of recompensation across different etiologies. Therefore, this study aimed to summarize differences in recompensation prevalence among decompensated cirrhosis etiologies according to the Baveno VII consensus, and to analyze the predictors and prognosis of recompensation.

**Methods:**

We screened four databases up to February 2, 2026, and included only cohort studies (prospective or retrospective) in English or Chinese. Quality was assessed using *the Joanna Briggs Institute Critical Appraisal Tool for prevalence studies*. A random‑effects model was used when I² > 50% and *p* < 0.05; otherwise, a fixed‑effects model was applied. We calculated pooled recompensation prevalence with 95% confidence intervals (CIs) for each etiology, evaluated predictors of recompensation, and assessed prognostic outcomes. Publication bias was assessed using Begg’s and Egger’s tests, and sensitivity analyses tested the robustness of the findings.

**Results:**

The total recompensation prevalence of decompensated cirrhosis was reported from 22 studies (*n* = 7585), with an overall recompensation prevalence of 34% (95%CI = 27%-41%), of which 38% (95%CI = 31%-45%) in the eastern region and 24% (95%CI = 9%-39%) in the western region. Hepatitis B virus (HBV)-related decompensated cirrhosis had the highest prevalence (49%, 95% CI = 33%-65%), followed by those with decompensated cirrhosis caused by hepatitis C virus (HCV), autoimmune liver disease (AILD), and alcoholic liver disease (ALD), with corresponding recompensation prevalence of 38% (95%CI = 23%-53%), 27% (95%CI = 24%-30%), 19% (95%CI = 3%-35%), respectively. Through pooled effect estimates, independent predictors of recompensation included gender, platelet count, albumin (ALB), Model for End-Stage Liver Disease (MELD) score, Child-Turcotte-Pugh (CTP) score, and Alanine Transaminase (ALT). For example, women and patients with higher ALB levels or lower MELD/CTP scores were more likely to achieve recompensation. Recompensation significantly improved the prognosis of patients with decompensated cirrhosis [hazard ratio (HR) = 2.81, 95%CI = 1.78–4.42, *p*<0.001].

**Conclusion:**

The prevalence of recompensation was highest in HBV-related decompensated cirrhosis, followed by HCV, then AILD, and lowest in ALD-related decompensated cirrhosis. Gender, platelet count, ALB, MELD score, CTP score, and ALT were independent predictors of recompensation. After achieving recompensation, the prognosis of patients with cirrhosis improved significantly. These findings suggest that etiology-specific recompensation prevalences may help guide individualized monitoring and treatment strategies in patients with advanced cirrhosis.

**Trial registration:**

CRD420261298240

**Supplementary Information:**

The online version contains supplementary material available at https://doi.org/10.1186/s12876-026-05087-8.

## Introduction

The compensated period of cirrhosis is the early stage of chronic and progressive liver disease caused by various etiologies (e.g., viral hepatitis, alcohol, cholestasis, drug toxicity). At this time, the liver has developed extensive fibrosis and regenerative nodules, but there are no specific clinical symptoms or signs. With apparent abnormalities in liver function and the occurrence of portal hypertension complications such as ascites, upper gastrointestinal bleeding, and hepatic encephalopathy, liver cirrhosis enters the decompensated period from the compensated period, accompanied by a significant deterioration in prognosis. Patients with compensated cirrhosis often live more than 12 years, while those with decompensated cirrhosis survive only 2–3 years on average [1].

In the past, the progression of cirrhosis from the compensated period to the decompensated period was long regarded as a point of no return. However, recent studies have shown that after effective etiological treatment, some patients with decompensated cirrhosis can be reversed to the compensated period, that is, to achieve “recompensation”. In 2021, the Baveno VII consensus proposed a unified definition of liver recompensation for the first time, namely: (1) clearance/inhibition/cure of the primary causes of cirrhosis, i.e., clearance of hepatitis C virus (HCV), continuous inhibition of hepatitis B virus (HBV), and continuous abstinence from alcohol for alcoholic cirrhosis; (2) Resolution of ascites (discontinuation of diuretics), resolution of hepatic encephalopathy (discontinuation of lactulose/rifaximin) and no recurrence of varicose vein bleeding for at least 12 months; (3) Liver function indicators albumin (ALB), international normalized ratio (INR) and bilirubin were stably improved [2]. Studies have shown that this recompensation significantly improves the survival prognosis of patients [3].

To date, the recompensation of decompensated cirrhosis and the Baveno VII consensus have been validated in several studies. However, prior studies were limited by single-disease focuses, inconsistent recompensation definitions, and a lack of pooled, cross-etiology comparisons. Considering the central position of etiological treatment in recompensation, the etiology of liver cirrhosis deserves our attention. A systematic review by Liu et al. [4] reported that, as of 2021, 42% of patients with cirrhosis worldwide had HBV infection and 21% had HCV infection, with an overall prevalence of HBV and HCV exceeding 50% in most parts of Asia and Africa. Another important cause is alcohol. The global prevalence of alcoholic liver disease (ALD)-related decompensated cirrhosis has also increased, from 25.3 per 100,000 cases in 1990 to 30 per 100,000 cases in 2017 [4]. In Europe, alcohol is the most common cause of advanced liver disease. In Asia, although HBV infection is still the leading cause of cirrhosis, the proportion of ALD-related cirrhosis has gradually increased in the past two decades, due to rising regional alcohol consumption and improved prevention/treatment of chronic viral hepatitis [5].

It is worth noting that a study in China [6] showed that the absolute number of cirrhosis cases from causes other than HBV, HCV, and alcohol use increased by nearly 50% from 1990 to 2016. These causes include nonalcoholic fatty liver disease (NAFLD), autoimmune liver disease (AILD), obesity, diabetes, and aflatoxin B1, etc., which may become a new primary concern for chronic liver disease in China.

Decompensated cirrhosis varies by etiology, with differences in pathogenesis, treatment, and efficacy, resulting in different recompensation outcomes [7]. Recompensation improves prognosis by reducing complications and mortality, and it guides treatment decisions by identifying which etiological subgroups are more likely to achieve clinical benefit. The dynamic changes in the etiological spectrum of liver cirrhosis make it difficult to ignore differences in treatment targets and disease outcomes across patient subgroups, and clarifying these differences is key to formulating individualized treatment strategies in clinical practice.

Therefore, this study aims to: (1) estimate the prevalence of recompensation according to Baveno VII criteria; (2) compare recompensation prevalences across different etiologies; (3) identify key predictors of recompensation; and (4) evaluate the prognostic impact of recompensation in patients with advanced cirrhosis, through a systematic review and meta-analysis.

## Research design and methods

### Literature search strategy

Two authors (JY Wang and MX Zhang) independently searched PubMed, Embase, Web of Science, and CNKI for relevant articles published from database inception to February 2, 2026. Given the limited number of relevant studies, the search strategy first broadens the scope and then screens full texts to identify relevant literature. The search terms used in different databases are shown in Supplementary Table 1. An example search strategy for PubMed is as follows: (cirrhosis [Title/Abstract] OR fibrosis [Title/Abstract]) AND recompensation [Title/Abstract]. The search results were first screened by title and abstract to exclude irrelevant literature, and then qualified studies were identified by reading the full texts. We also manually screened the reference lists of the included studies to identify any additional eligible studies. Disagreements regarding study inclusion were discussed with the third author (SH Tang) and resolved by consensus. This study conducted a systematic review using a specified protocol in accordance with the Preferred Reporting Items for Systematic Reviews and Meta-Analyses (PRISMA) statement [8]. Our review is registered in the PROSPERO database (registration number: CRD420261298240) [9].

### Literature inclusion/exclusion criteria

We included studies that met the following criteria: (1) enrolled adults with decompensated cirrhosis; (2) were cohort studies (prospective or retrospective); (3) reported recompensation according to the Baveno VII criteria. Only studies published in English or Chinese were included. Reviews, systematic reviews, meta-analyses, conference abstracts, and duplicate publications were excluded. If multiple studies used the same patient cohort, only the most comprehensive report was included.

### Predictor analysis

All studies included in the predictor analysis had consistent definitions for each predictor and comparable effect measures. Only predictors reported in at least two studies were considered for pooling. Pooling these studies increases statistical power and provides more precise estimates than any single study alone, thereby strengthening the evidence for each predictor.

### Data extraction and literature quality evaluation

Two researchers (JY Wang and MX Zhang) independently extracted data using a standardized data extraction form, which included the following items: the first author’s name, publication year, country, study design, etiology of cirrhosis, total sample size, number of patients with recompensation, basic characteristics of patients, prognostic data [such as hazard ratio (HR)], predictors of analysis, etc. For the correlation analysis of predictors, the results of the multivariate analysis were extracted preferentially. If not provided by the study, the univariate analysis results were extracted. We did not contact the authors of the original studies for missing data, as all necessary information was available from the published reports. Two authors (JY Wang and MX Zhang) independently evaluated the methodological quality of the included studies using the Joanna Briggs Institute (JBI) Critical Appraisal Tool for prevalence studies [10]. Studies with a score of 7–9 were considered high quality, 4–6 medium quality, and 1–3 low quality. Only studies scoring ≥ 7 (high quality) were included (Table 1). Disagreements during data extraction or quality assessment were resolved by discussion between the two authors (JY Wang and MX Zhang) or by consulting a third author (SH Tang).

### Statistical methods

We calculated the combined value of the overall recompensation prevalence and the recompensation prevalence for different subgroups using the STATA 19.0 command metan and reported it with 95% CIs. For proportion meta‑analysis, we used the normal approximation method without transformation (p = n/N, se = √[p(1-p)/N]), which is appropriate when proportions are not extreme. When reporting the predictors of recompensation, the odds ratio (OR) and 95% CI are reported; when reporting the prognosis of patients with recompensation, the HR and 95% CI are reported, and *P* < 0.05 is considered a statistically significant difference. For ORs and HRs, we pooled the log‑transformed values (log OR and log HR) using the inverse‑variance method, and exponentiated the results to obtain pooled ORs and HRs with 95% CIs. We assessed heterogeneity between studies using Cochran’s Q statistic and I² statistic. If significant heterogeneity was found between studies (I^2^ >50%, *P* < 0.05), the random-effects model was used; otherwise, the fixed-effects model was used. Begg’s test and Egger’s test were used to evaluate publication bias, and *P* < 0.05 indicated the existence of potential publication bias. We conducted a sensitivity analysis to assess the robustness of the summary results. Meta‑regression was not planned because our primary objective was to compare recompensation prevalences across predefined etiological subgroups using subgroup analysis, which is appropriate for categorical variables. No zero‑event studies were identified in our included studies.

## Results

**Fig. 1:**
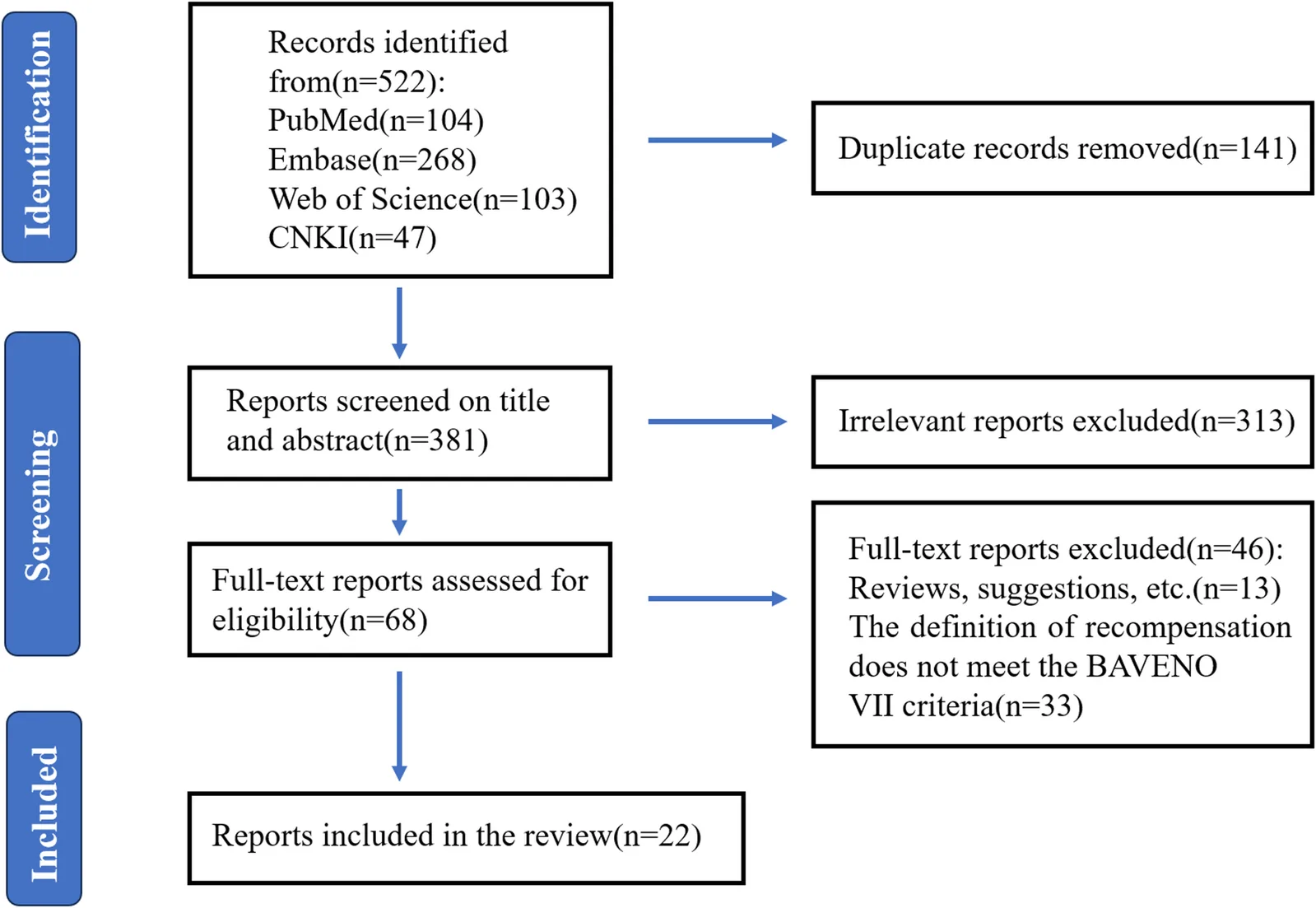
Systematic reviews and meta-analyses flow diagram of the search strategy

According to the search strategy, we screened and systematically analyzed articles related to recompensation in decompensated cirrhosis. Of 522 initially identified articles, 22 cohort studies (7,585 participants; 18 in English, 4 in Chinese) met the inclusion criteria after duplicate removal, title/abstract screening, and full‑text review, etc. (Fig. 1). Details of all included studies are shown in Table 1. The detailed list of recompensation predictors reported in each study is provided in Supplementary Table 2.

**Table 1:** Characteristics of included studies

Author & Year	Region	Study type	Etiology	Recompensation, (*n*)/Decompensated cirrhosis(*N*)	Recompensation predictors reported	Factor effect index	Survival HR (95% CI)	JBI score
Gao L, et al.2023[11]	China	retrospective study	all-cause	20/64	YES	OR	NA	7
He Z, et al.2023[12]	China	retrospective study	HBV	203/383	NO	NA	NA	7
Hui VW, et al.2023[13]	China	retrospective study	HBV	265/1374	NO	NA	NA	8
Hofer BS, et al.2023[3]	Europe	retrospective study	ALD	37/204	YES	HR	NA	8
Deng Y, et al.2024[14]	China	retrospective extension of a prospective study	HBV	159/283	YES	OR	NA	8
Hofer BS, et al.2024[15]	Europe	retrospective study	PBC	7/42	YES	HR	2.174(0.452,10.000)	7
Premkumar M, et al.2024[16]	India	prospective study	HCV	284/1152	YES	HR	1.724(1.124,2.703)	8
Yang S, et al.2024[17]	China	retrospective study	all-cause	38/89	YES	HR	NA	7
Zhang Y, et al.2024[18]	China	retrospective study	HBV	100/171	YES	HR	NA	8
Bai Y, et al.2025[19]	China	retrospective study	all-cause	126/621	YES	OR	2.275(1.606,3.223)	7
Lin H, et al.2025[20]	China	retrospective study	PBC	105/401	YES	HR	NA	8
Xian M, et al.2025[21]	China	retrospective study	HBV	134/360	YES	OR	NA	7
Chen Y, et al.2025[22]	China	retrospective study	AIH	68/220	YES	HR	20.510(2.790,150.630)	8
Ding D, et al.2025[23]	China	retrospective study	PBC	45/170	YES	HR	16.480(2.230,121.570)	8
Sánchez-Torrijos Y, et al.2025[24]	Europe	retrospective study	HCV	137/216	YES	OR	3.333(2.041,5.263)	7
Semmler G, et al.2025[25]	Europe	retrospective study	HCV	132/361	YES	HR	NA	7
Tonon M, et al.2025[26]	Europe	retrospective study	HBV/HCV/ALD	21/525	YES	HR	NA	8
Xu D, et al.2025[27]	China	retrospective study	HCV	63/217	YES	OR	NA	8
Xu Y, et al.2025[28]	China	retrospective study	HBV	80/136	YES	OR	NA	8
Yin D, et al.2025[29]	China	retrospective study	HBV	99/166	YES	OR	NA	8
Zanetto A, et al.2025[30]	Europe	retrospective study	ALD	12/224	YES	HR	NA	8
Zhou JY, et al.2025[31]	China	retrospective study	ALD	70/206	YES	OR	NA	8

*HBV* Hepatitis B virus, *HCV* Hepatitis C virus, *PBC* Primary biliary cholangitis, *AIH* Autoimmune hepatitis, *ALD* Alcoholic liver disease, *OR* Odds ratio, *HR* Hazard ratio, *CI* Confidence interval, *JBI* Joanna Briggs Institute

### The overall recompensation prevalence of decompensated cirrhosis and the difference in the prevalence of recompensation in different subgroups

**Fig. 2:**
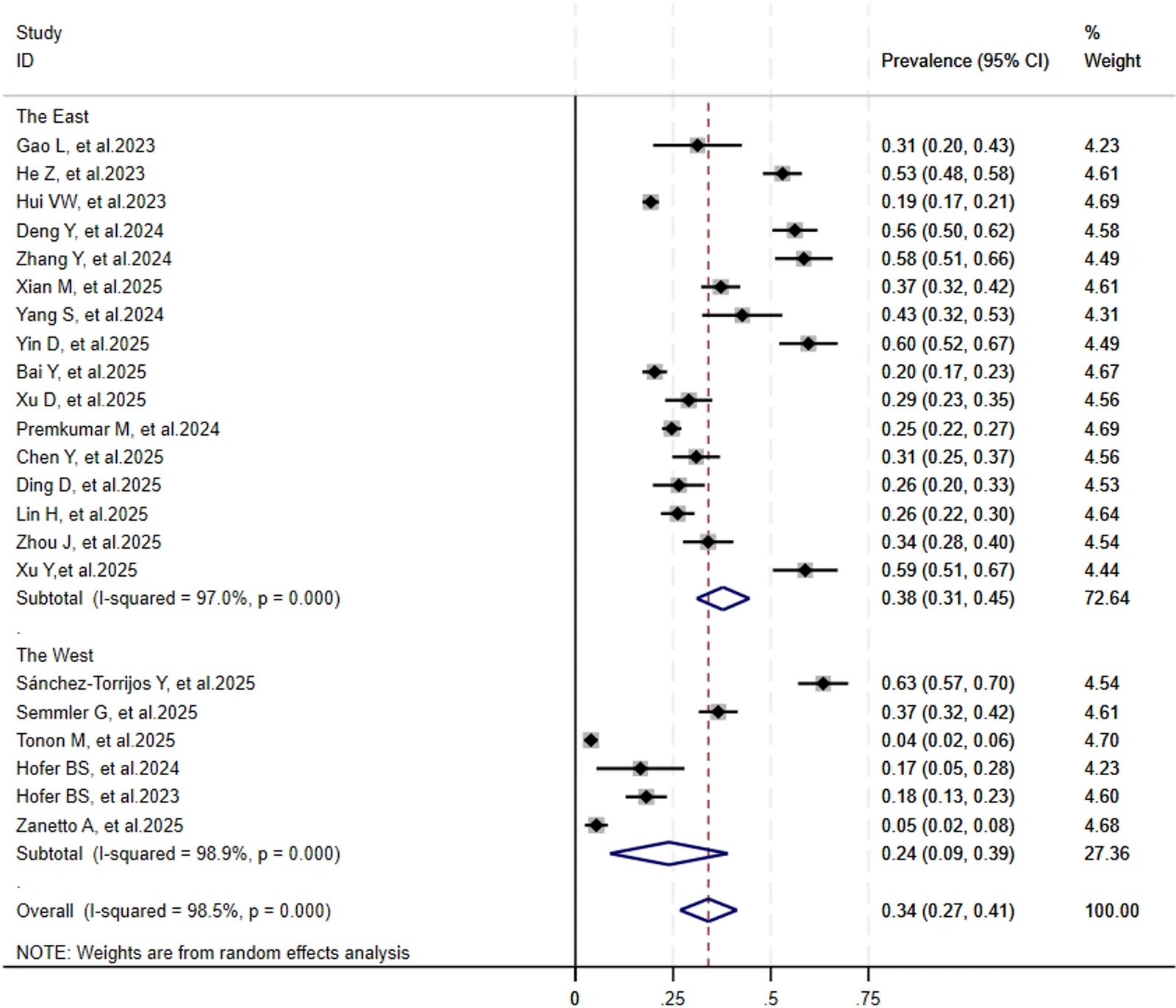
Overall recompensation prevalence of patients with decompensated cirrhosis and stratified by geography

**Fig. 3:**
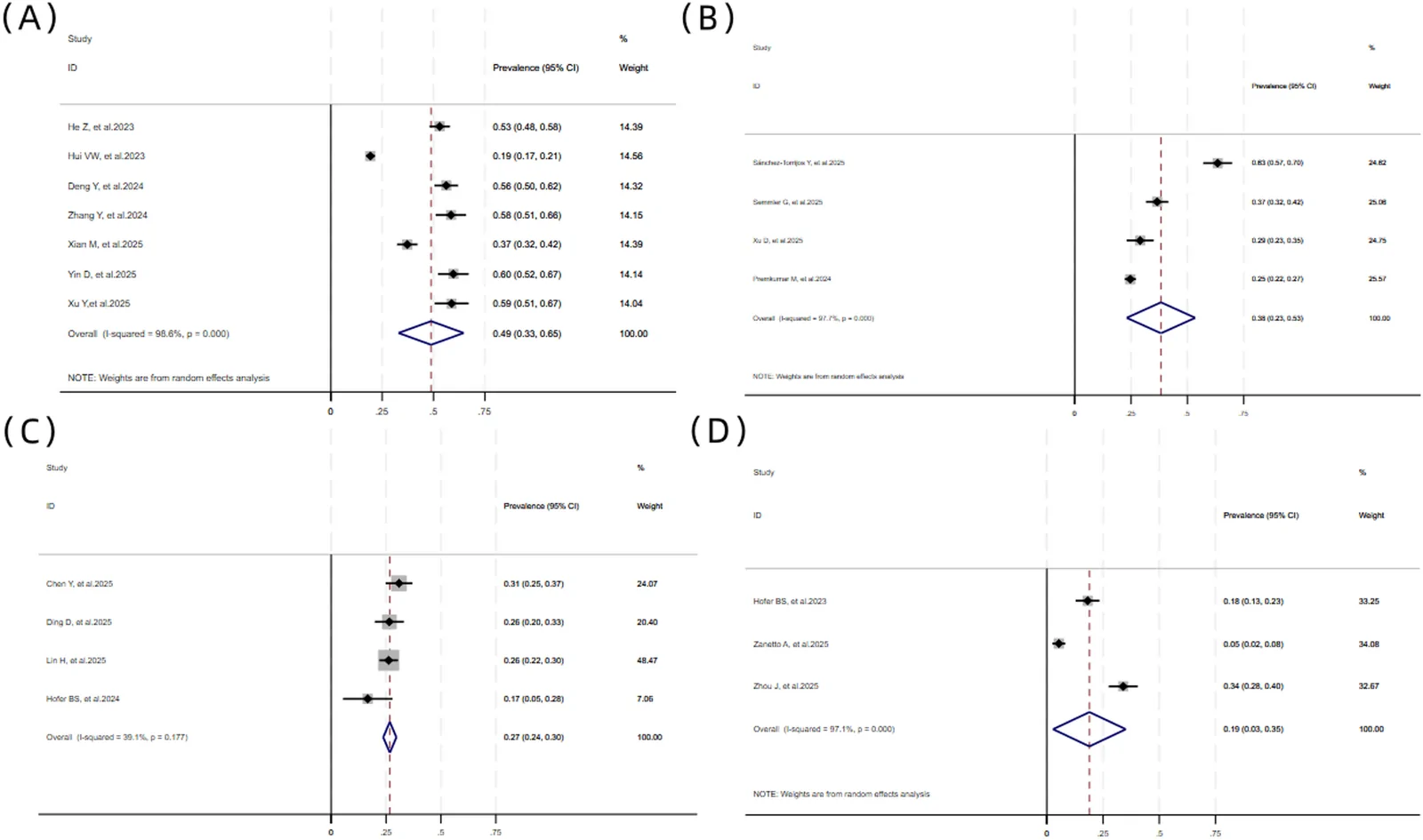
Recompensation prevalence in patients with decompensated cirrhosis across different etiological subgroups. **A** HBV-related decompensated cirrhosis; (**B**) HCV-related decompensated cirrhosis; (**C**) AILD-related decompensated cirrhosis; (**D**) ALD-related decompensated cirrhosis

The included studies were from China (*n* = 15), India (*n* = 1), and Europe (*n* = 6: 3 from Austria, 2 from Italy, and 1 from Spain). The recompensation prevalence of decompensated cirrhosis was reported from these 22 studies, with a total prevalence of recompensation of 34% (95% CI = 27% − 41%, *p* < 0.001), 38% (95% CI = 31% − 45%, *p* < 0.001) in the East (China, India), and 24% (95% CI = 9%-39%, *p* = 0.002) in the West, as shown in Fig. 2. Subgroup analyses of 18 single-etiology studies (*n* = 6,286) showed the following recompensation prevalences (Fig. 3): HBV-related decompensated cirrhosis had the highest prevalence (49%, 95% CI: 33%–65%, *p* < 0.001). HCV-related cirrhosis had a prevalence of 38% (95% CI: 23%–53%, *p* < 0.001). AILD-related cirrhosis had a prevalence of 27% (95% CI: 24%–30%, *p* < 0.001). ALD-related cirrhosis had the lowest prevalence (19%, 95% CI: 3%–35%, *p* = 0.022).

Significant heterogeneity was observed in the meta-analysis of the overall recompensation prevalence (I²=98.5%, *p* < 0.001, Fig. 2). The sensitivity analysis showed that the pooled effect size remained stable between 32% and 36% (original pooled rate: 34%, Supplementary Fig. 1).

Significant heterogeneity was also observed across most subgroup analyses (I² > 97%), except for the AILD subgroup (I² = 39.1%). In the analysis by regional subgroup, the combined effect size in the Eastern population ranged from 36% to 40% (the original pooling rate was 38%) after removing the literature one by one, suggesting that the results were relatively stable. The combined effect size in the Western population ranged from 16% to 28% (the original pooling rate was 24%), and the pooling rate decreased to 16% after excluding Sánchez-Torrijos et al. [24]. After excluding Tonon et al. [26] and Zanetto et al. [30], the 95% CI expanded to 7%-49% and 6%-50%, respectively. The CIs of all exclusion analyses overlapped with the original intervals, and no single study was found to have a subversive effect on the overall conclusion (Supplementary Fig. 2).

In the analysis by etiological subgroup, the combined effect size for the HBV subgroup ranged from 47% to 54% (the original pooled rate was 49%). After excluding Hui et al. [13], the pooling rate increased to 54%, the 95% CI narrowed to 46%- 61%, and the results of the remaining exclusion analyses were highly consistent with the overall pooled results, suggesting stability. The pooled effect size ranged from 30% to 43% in the HCV subgroup (the original pooled rate was 38%). After excluding Sánchez-Torrijos et al. [24], the pooling rate dropped to 30%, the 95% CI narrowed to 22%-38%, and the CIs of all exclusion analyses overlapped with the original intervals, with no disruptive effects. The original pooled effect rate of the ALD subgroup was 19%, and the fluctuation range was 11%-26% after excluding one by one. This subgroup has few included literature and limited stability of results, so it needs to be interpreted with caution (Supplementary Fig. 3).

The Begg and Egger tests were used to assess publication bias in the included studies, as shown in Table 2. Egger’s test was significant for some comparisons (*p* < 0.001), but its interpretation for proportion meta‑analyses is limited, and it may be overly sensitive when study numbers are small or heterogeneity is high.

**Table 2:** Publication bias in the meta-analysis of the prevalence of recompensation in patients with decompensated cirrhosis

Combined Effect Size	Studies	Decompensated cirrhosis(*N*)	Recompensation (*n*)	Begg’s	Egger’s
Overall prevalence [3, 11–31]	22	7585	2205	*p* = 0.080	*p*<0.001
Eastern prevalence [11–14, 16–23, 27–29, 31]	16	6013	1859	*P* = 0.079	*P* = 0.001
Western prevalence [3, 15, 24–26, 30]	6	1572	346	*P* = 0.260	*P* = 0.092
HBV-related prevalence [12–14, 18, 21, 28, 29]	7	2873	1040	*P* = 0.764	*P* = 0.001
HCV-related prevalence [16, 24, 25, 27]	4	1946	616	*P* = 0.308	*P* = 0.229
AILD-related prevalence [15, 20, 22, 23]	4	833	225	*P* = 0.734	*P* = 0.480
ALD-related prevalence [3, 30, 31]	3	634	119	*P* = 0.296	*P* = 0.137

*HBV* Hepatitis B virus, *HCV* Hepatitis C virus, *AILD* Autoimmune liver diseases, *ALD* Alcoholic liver disease

### Predictors and prognosis of recompensation in patients with decompensated cirrhosis

Sixteen studies have explored the predictors of recompensation in decompensated cirrhosis patients (Table 3), among which Hofer BS et al. [15] and Zanetto A et al. [30] performed only univariate analysis. Among the predictors, gender was a binary variable, and baseline bilirubin, platelets, ALB, Child-Turcotte-Pugh (CTP) score, Model for End-Stage Liver Disease (MELD) score, Body Mass Index (BMI), Alpha-fetoprotein (AFP), and Alanine Transaminase (ALT) were continuous variables. Gender (OR for males vs. females = 0.302, 95%CI: 0.165–0.553, *p* < 0.001), platelets (OR = 1.008, 95%CI: 1.000-1.016, *p* = 0.045), ALB (OR = 1.120, 95%CI: 1.045-1.200, *p* = 0.001), MELD score (OR = 0.852, 95%CI: 0.781–0.931, *p* < 0.001), CTP score (OR = 0.717, 95%CI: 0.612–0.840, *p* < 0.001) and ALT (OR = 1.010, 95%CI: 1.006–1.014, *p* < 0.001) had a significant impact on the recompensation of decompensated cirrhosis (Supplementary Fig. 4). For example, women were more likely to achieve recompensation.

**Table 3:** Meta-analysis of predictors of recompensation

Predictors	Combination studies	Heterogeneity of study design		Analysis model	Results of meta-analysis	
		I^2^	*P*		OR (95%CI)	*P*
Gender [18, 26]	2	0.0%	0.902	Fixed	0.302(0.165, 0.553)	<0.001
TBIL [14–16, 20, 22, 23]	6	87.1%	<0.001	Random	0.959(0.907, 1.013)	0.136
Platelet [19, 22, 23, 30]	4	77.8%	0.004	Random	1.008(1.000, 1.016)	0.045
ALB [21, 25, 26]	3	73.8%	0.022	Random	1.120(1.045, 1.200)	0.001
MELD score [26, 30]	2	28.6%	0.237	Fixed	0.852(0.781, 0.931)	<0.001
CTP score [3, 15, 24]	3	0.0%	0.497	Fixed	0.717(0.612, 0.840)	<0.001
BMI [20, 26]	2	88.2%	0.004	Random	0.938(0.722, 1.219)	0.633
AFP [21, 27]	2	80.1%	0.025	Random	1.007(0.998, 1.016)	0.126
ALT [18, 28]	2	0.0%	1.000	Fixed	1.010(1.006, 1.014)	<0.001

*TBIL* Total bilirubin, *ALB* Albumin, *MELD* Model for end-stage liver disease, *CTP* Child-Turcotte-Pugh, *BMI* Body mass index, *AFP *Alpha-fetoprotein, *ALT *Alanine transaminase, *OR* Odds ratio, *CI *Confidence interval

**Fig. 4:**
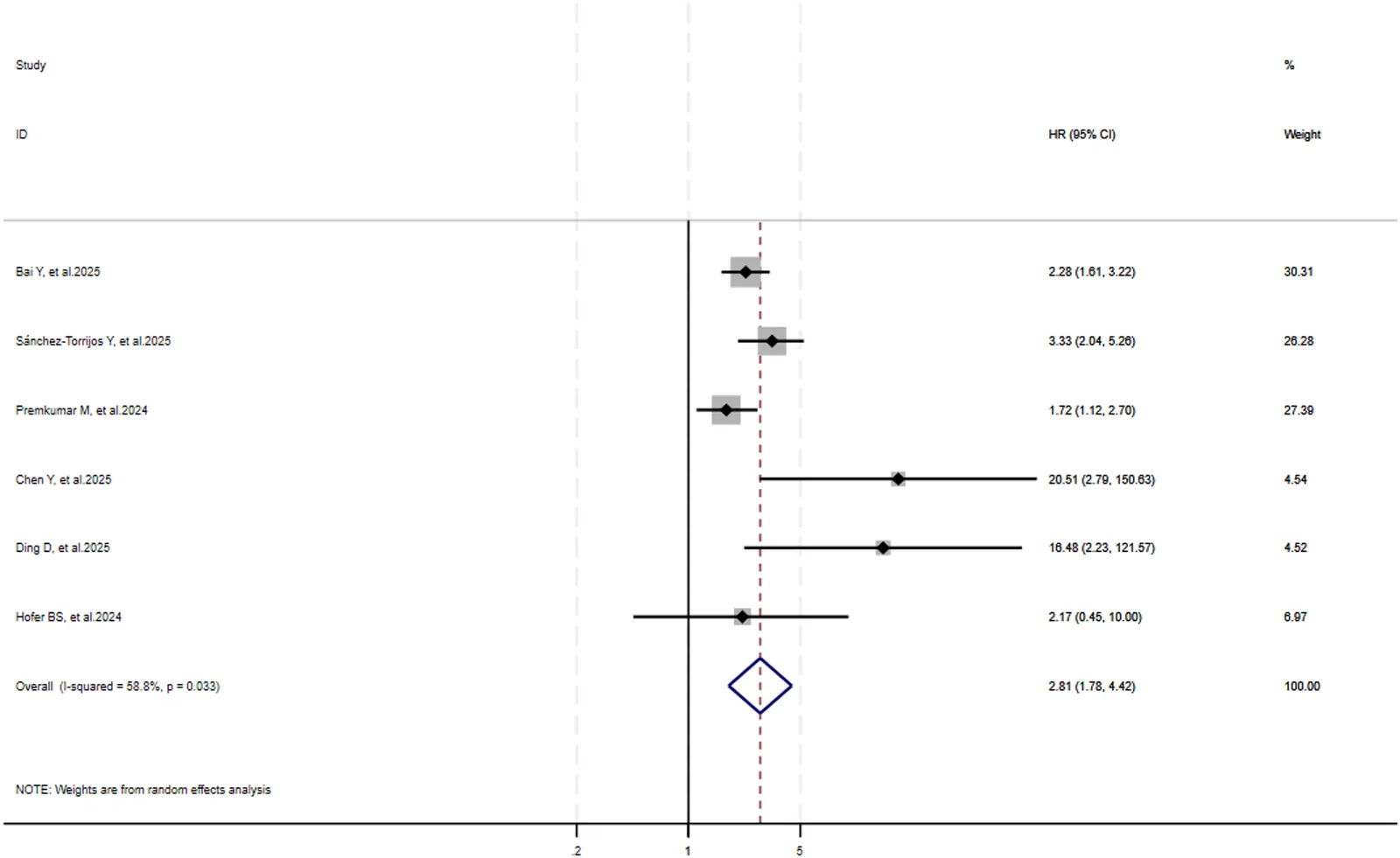
Impact of recompensation on prognosis in patients with decompensated cirrhosis (non‑recompensation vs. recompensation)

Six studies (*n* = 2421) compared the prognosis between patients with and without recompensation after decompensated cirrhosis and provided HR. Pooled effect sizes revealed a significant difference between the two groups (non‑recompensation group vs. recompensation group: HR = 2.81, 95%CI: 1.78–4.42, *p* < 0.001; Fig. 4), indicating that patients who did not achieve recompensation had nearly three times the risk of poor prognosis compared to those who achieved recompensation. The Begg’s and Egger’s tests showed no significant publication bias (Begg’s: *p* = 0.133; Egger’s: *p* = 0.133), and the sensitivity analysis showed stable results (Supplementary Fig. 5).

## Discussion

This is the first meta‑analysis to compare recompensation prevalence across different etiologies using the standardized Baveno VII criteria. The primary finding is that recompensation prevalences vary across etiologies, with the highest observed in HBV‑related cirrhosis and the lowest in ALD‑related cirrhosis. It is worth noting that the recompensation prevalence in the ALD subgroup fluctuates widely, so these results should be interpreted with caution. In the regional subgroup, the overall recompensation prevalence of Eastern patients was higher than that of Western patients, which may be attributed to differences in the etiological spectrum of cirrhosis between Eastern and Western populations rather than a true regional effect. Individual patient data were not available to formally adjust for this confounding. In Europe, alcohol is the most common cause of advanced liver disease, whereas HBV infection is the leading etiology in Asia. Notably, a sensitivity analysis of Western patient subgroups shows that results fluctuate widely, and the true differences between the East and the West need to be further clarified.

Advanced cirrhosis of different causes responds differently to etiological treatment. For HBV infection, nucleoside or nucleotide analogue (NUC) therapies are the preferred option because they can reliably suppress viral load with few side effects [32, 33]. Unlike immunomodulators (e.g., interferon), which can cause significant side effects, NUCs are well tolerated and suitable for long‑term use. Studies have shown that successful antiviral therapy can reverse HBV‑induced liver fibrosis and cirrhosis, reduce liver inflammation, and create conditions for hepatocyte regeneration and functional recovery [34, 35]. This may explain why HBV‑related decompensated cirrhosis had the highest recompensation prevalence (49%) among all etiologies in our study. Clinically, this finding supports the use of timely and sustained NUC therapy in these patients to maximize the chance of achieving recompensation.

HCV infection has been transformed by the introduction of oral direct‑acting antivirals (DAAs), which cure nearly all patients regardless of genotype or fibrosis stage, with few side effects [33]. Achieving sustained virologic response (SVR) is the primary goal of HCV treatment. Hanafy et al. [36] showed that after treatment with DAAs, patients with HCV-related decompensated cirrhosis and adequate liver volume had an SVR of 90%, and CTP score, MELD score, and survival were all improved. Soliman et al. [37] found that DAAs can also significantly reduce liver fibrosis in patients. Unlike HBV, which requires long‑term suppression, DAAs can completely eradicate HCV. Despite this virologic cure, our study found that HCV‑related decompensated cirrhosis had a lower recompensation prevalence (38%) than HBV‑related cirrhosis (49%). A possible reason is that HCV infection progresses slowly with insidious symptoms, leading to delays in diagnosis and treatment.

AILD includes autoimmune hepatitis (AIH), primary biliary cholangitis (PBC), and primary sclerosing cholangitis (PSC). For AIH, standard treatment is immunosuppressive therapy (IST) consisting of prednisolone alone or in combination with other immunosuppressants. The therapeutic endpoint is a complete biochemical response [CBR, normalization of transaminase and immunoglobulin G (IgG)], which is key to improving patient outcomes [38]. Chen et al. [22] reported that AIH‑related decompensated cirrhosis patients who achieved CBR had a significantly higher prevalence of recompensation than those without CBR. However, a substantial proportion of AIH patients do not achieve CBR. In a large national study, the biochemical response rate was only 59% [39]. PBC is a chronic autoimmune cholestatic liver disease treated primarily with ursodeoxycholic acid (UDCA). Elevated alkaline phosphatase (ALP) and bilirubin are important clinical markers in PBC [40, 41]. Only about 60% of patients respond adequately to UDCA, while the remaining 40% have persistent biochemical abnormalities and reduced transplant‑free survival [42]. In a study of PBC‑associated decompensated cirrhosis, 36% of patients met the Paris II response criteria (i.e., normalization of bilirubin and a decrease in ALP to ≤ 1.5 times the upper limit of normal) after one year of UDCA therapy [15]. Unlike HBV and HCV, where etiological treatment directly targets the cause of liver injury, treatment for AILD controls inflammation but does not correct the underlying immune disorder. Consequently, some patients have persistent biochemical abnormalities (e.g., elevated ALP or bilirubin), and the liver remains in a state of ongoing injury. This likely explains why our study found that AILD‑related decompensated cirrhosis had a lower recompensation prevalence (27%) than HBV (49%) or HCV (38%) cirrhosis.

For ALD‑related cirrhosis, there is currently a lack of specific treatments. The cornerstone of management is sustained abstinence from alcohol, which is associated with significant improvements in patient outcomes. A meta‑analysis by Lim et al. [43] (19 studies, 18,833 patients) showed that approximately 52.7% of patients with decompensated alcohol‑related cirrhosis were able to abstain from alcohol. During a mean follow‑up of 48.6 months, survival was significantly lower in those who continued drinking. Despite this, our study found that ALD‑related cirrhosis had the lowest recompensation prevalence (19%) among all etiologies. Possible reasons include: (1) unlike viral hepatitis (controlled by drugs) or AILD (inflammation suppressed), the only effective treatment for ALD is behavioral (abstinence), which is highly dependent on patient compliance and less controllable; (2) alcohol and its metabolites cause multi‑system damage (e.g., hepatotoxicity, gut dysbiosis, nutritional disorders), weakening liver regenerative capacity; (3) alcoholic cirrhosis is often diagnosed at a more advanced stage, with more severe fibrosis and structural damage.

The meta‑analysis of predictors of recompensation showed that gender, platelet count, ALB, MELD score, CTP score, and ALT were independent predictors. Women were more likely to achieve recompensation than men, consistent with previous studies. Higher platelet counts and ALB levels, as well as lower MELD and CTP scores, were associated with higher recompensation odds, reflecting better liver functional reserve and regenerative capacity. The finding that higher ALT levels predicted recompensation (OR = 1.010) appears paradoxical, as ALT is typically a marker of hepatocyte injury. However, this ‘ALT paradox’ may reflect treatment‑responsive inflammation: in patients with HBV‑related cirrhosis (the only etiology in the studies reporting ALT [18, 28]), higher ALT levels may indicate active hepatic necroinflammation that responds better to antiviral therapy, leading to subsequent recompensation. The small effect size (OR close to 1) suggests that ALT is a weak predictor and should be interpreted cautiously. Given the limited literature, this study did not explore differences in predictors across etiologies. Future research with larger sample sizes is needed to refine etiology‑specific management.

The prognostic impact of recompensation was strong: patients who did not achieve recompensation had nearly three times the risk of poor outcomes compared to those who achieved recompensation (HR = 2.81). The combined results were derived from 6 studies (*n* = 2421), including 3 on AILD (2 on PBC and 1 on AIH), 2 on HCV, and 1 on all causes. In these 6 studies, Bai et al. [19] included the population as all-cause, with the majority (58.1%) having HBV-related cirrhosis, and the other causes included HCV, alcohol, schistosomiasis, AIH, etc. Due to small sample sizes, etiology‑specific prognosis could not be analyzed. This still requires multicenter, large-sample studies to explore differences in prognosis after recompensation in decompensated cirrhosis of different etiologies, to help refine disease management.

Compared with a prior meta‑analysis (27 studies, 9,063 patients) [44], both studies found HBV to have the highest recompensation rate (50% vs. 49%). However, for ALD, the prior study reported a rate of 33%, which was higher than PBC (15%) and AIH (25%), whereas our study found ALD to have the lowest rate (19%). Our study advances the field by uniformly applying the Baveno VII criteria across all etiologies, whereas the prior meta‑analysis did not restrict inclusion to this standardized definition.

There are still several limitations in this study. First, although all included studies used a uniform definition of recompensation and stratified analyses by etiology, substantial heterogeneity (I^2^ > 97%) was observed across all subgroups except the AILD group. This suggests that, even with control for definition and etiological factors, confounding factors such as baseline liver function reserve, number of decompensations, type and number of complications, degree of portal hypertension, treatment regimen, patient selection and compliance, regional differences in healthcare practices, and follow-up time remain difficult to eliminate. Due to limitations in the original study data, the above factors cannot be further corrected, resulting in limited stability in the combined results for some subgroups, especially the ALD group, which should be interpreted with caution. Further analysis should be performed in future studies with larger sample sizes. Secondly, only 4 databases were searched in this study, potentially leading to the omission of relevant studies and data. Third, the majority of included studies were from China, which limits the generalizability of our findings to other populations with different etiological spectra and healthcare practices. Fourth, the Egger’s test showed significant publication bias in some studies. It should be noted that the prevalence of recompensation is a descriptive outcome rather than an effect-size comparison, so the publication bias test is of little significance; this bias is more likely to reflect high heterogeneity between studies or the absence of unpublished negative or null findings. Fifth, in the meta‑analysis of predictors, several limitations should be noted. The number of studies for each predictor was very small. Multivariate estimates were extracted preferentially; when not available, univariate estimates were used. Both ORs and HRs from original studies were pooled as ORs due to the limited data. These limitations reduce the robustness of the predictor findings, and the results should be interpreted cautiously. Sixth, some of the included studies are small, which is prone to bias and undermines the reliability of the conclusions.

## Conclusion

In conclusion, the prevalence of recompensation varies among patients with decompensated cirrhosis of different etiologies under the Baveno VII criteria. Among them, HBV-related decompensated cirrhosis had the highest prevalence, followed by patients with HCV and AILD (AIH/PBC), and ALD-related decompensated cirrhosis had the lowest prevalence. Gender, platelet count, ALB, MELD score, CTP score, and ALT were independent predictors of decompensated cirrhosis. After achieving recompensation, the prognosis of patients with cirrhosis was significantly improved. Identifying etiological differences in recompensation can help guide individualized patient monitoring and treatment strategies to improve prognosis.

## Supplementary Information

Supplementary Material 1.

Supplementary Material 2.

Supplementary Material 3.

## Data Availability

The datasets used and/or analysed during the current study are available from the corresponding author on reasonable request.

## Abbreviations

CI: Confidence interval
HBV: Hepatitis B virus
HCV: Hepatitis C virus
AILD: Autoimmune liver disease
PBC: Primary biliary cholangitis
AIH: Autoimmune hepatitis
ALD: Alcoholic liver disease
CTP: Child-Turcotte-Pugh
TBIL: Total bilirubin
MELD: Model for end-stage liver disease
ALP: Alkaline phosphatase
UDCA: Ursodeoxycholic acid
INR: International normalized ratio
ALT: Alanine transaminase
BMI: Body mass index
AFP: Alpha-fetoprotein
ALB: Albumin
IgG: Immunoglobulin G
OR: Odds ratio
HR: Hazard ratio
JBI: Joanna Briggs Institute
