# Supplementary material for: Etiology-related differences in hepatic recompensation in decompensated cirrhosis under Baveno VII criteria: a systematic review and meta-analysis
Source: BMC Gastroenterol. 2026 Jul 8;26:595. doi: 10.1186/s12876-026-05087-8 (PMC13629081; doi:10.1186/s12876-026-05087-8)
Supplement: Supplementary file 1 [file 12876_2026_5087_MOESM1_ESM.docx]

**Supplementary Table 1. Different databases and corresponding search strategies**

| **Databases** | **Queries** |
| --- | --- |
| PubMed | (cirrhosis [Title/Abstract] OR fibrosis [Title/Abstract]) AND recompensation [Title/Abstract] |
| Embase | (cirrhosis: ti, ab, kw OR fibrosis: ti, ab, kw) AND recompensation: ti, ab, kw |
| Web of Science | ((cirrhosis OR fibrosis) AND recompensation (Title)) or ((cirrhosis OR fibrosis) AND recompensation (Abstract)) or ((cirrhosis OR fibrosis) AND recompensation (Author Keywords)) |
| CNKI | ((Title, Keyword and Abstract: cirrhosis) OR (Title, Keyword and Abstract: fibrosis)) AND (Title, Keyword, and Abstract: recompensation) |

**Supplementary Figure 1. Sensitivity analysis of the overall recompensation prevalence in decompensated cirrhosis**

**Supplementary Figure 2. (A) Sensitivity analysis of the recompensation prevalence in Eastern patients with decompensated cirrhosis; (B) Sensitivity analysis of the recompensation prevalence in Western patients with decompensated cirrhosis**

**Supplementary Figure 3. Sensitivity analysis of the recompensation prevalence in patients with decompensated cirrhosis across different etiological subgroups. (A) HBV-related decompensated cirrhosis; (B) HCV-related decompensated cirrhosis; (C) AILD-related decompensated cirrhosis; (D) ALD-related decompensated cirrhosis**

**Supplementary Figure 4. The forest plot summarizes the odds ratios for predictors associated with recompensation in decompensated cirrhosis. (A) Gender; (B) Platelet; (C) Albumin; (D) MELD; (E) CTP; (F)ALT**

**Supplementary figure 5. Sensitivity analysis of prognostic hazard ratio after recompensation in decompensated cirrhosis**
