# Supplementary material for: Etiology-related differences in hepatic recompensation in decompensated cirrhosis under Baveno VII criteria: a systematic review and meta-analysis
Source: BMC Gastroenterol. 2026 Jul 8;26:595. doi: 10.1186/s12876-026-05087-8 (PMC13629081; doi:10.1186/s12876-026-05087-8)
Supplement: Supplementary file 3 [file 12876_2026_5087_MOESM3_ESM.docx]

**Supplementary Table 1. Different databases and corresponding search strategies**

| **Databases** | **Queries** |
| --- | --- |
| PubMed | (cirrhosis [Title/Abstract] OR fibrosis [Title/Abstract]) AND recompensation [Title/Abstract] |
| Embase | (cirrhosis: ti, ab, kw OR fibrosis: ti, ab, kw) AND recompensation: ti, ab, kw |
| Web of Science | ((cirrhosis OR fibrosis) AND recompensation (Title)) or ((cirrhosis OR fibrosis) AND recompensation (Abstract)) or ((cirrhosis OR fibrosis) AND recompensation (Author Keywords)) |
| CNKI | ((Title, Keyword and Abstract: cirrhosis) OR (Title, Keyword and Abstract: fibrosis)) AND (Title, Keyword, and Abstract: recompensation) |

**Supplementary Table 2. Characteristics of included studies with recompensation predictors**

| **Author & Year** | **Recompensation predictors reported** | | |
| --- | --- | --- | --- |
| Gao L, et al.2023[11] | Age, portal venous pressure gradient < 12 mmHg after TIPS |  |  |
| He Z, et al.2023[12] | NA | |  |
| Hui VW, et al.2023[13] | NA | |  |
| Hofer BS, et al.2023[3] | CTP score, MAP | |  |
| Deng Y, et al.2024[14] | HBV DNA, DBIL, Na^＋^, TBIL | |  |
| Hofer BS, et al.2024[15] | MELD‑Na, CTP score, TBIL, ALP, GGT, UDCA response at 12 months | |  |
| Premkumar M, et al.2024[16] | TBIL, INR, Presence of large EV, Presence of GV, Adherence | |  |
| Yang S, et al.2024[17] | MELD score < 19, early TIPS (< 72 hours) | |  |
| Zhang Y, et al.2024[18] | Age, gender, ALT | |  |
| Bai Y, et al.2025[19] | Sarcopenia, PLT | |  |
| Lin H, et al.2025[20] | LMR, BMI, TBIL | |  |
| Xian M, et al.2025[21] | HE， NLPR， AFP， ALB | |  |
| Chen Y, et al.2025[22] | IgG, TBIL, PLT, Ascites only | |  |
| Ding D, et al.2025[23] | PLT, TBIL | |  |
| Sánchez-Torrijos Y, et al.2025[24] | CTP score, the number of past decompensating events | |  |
| Semmler G, et al.2025[25] | Diabetes, ALB | |  |
| Tonon M, et al.2025[26] | Gender, BMI, Hemoglobin, MELD score, ALB, Further  decompensation | |  |
| Xu D, et al.2025[27] | History of HIV infection, Ascites grade, PSE, AFP, Portal vein diameter | |  |
| Xu Y, et al.2025[28] | ALT, ALT/logqHBsAg＞23.48 | |  |
| Yin D, et al.2025[29] | APRI | |  |
| Zanetto A, et al.2025[30] | MELD score, CTP score C, presepsin, PLT | |  |
| Zhou JY, et al.2025[31] | NLR, PVD, CHE | |  |

HBV, Hepatitis B virus; TIPS, Transjugular intrahepatic portosystemic shunt; CTP, Child-Turcotte-Pugh; MAP, Mean arterial pressure; Na^＋^, Sodium; DBIL, Direct bilirubin; TBIL, Total bilirubin; MELD, Model for end-stage liver disease; MELD‑Na, Model for end-stage liver disease-sodium; ALP, Alkaline phosphatase; GGT, Gamma-glutamyl transferase; UDCA, Ursodeoxycholic acid; INR, International normalized ratio; EV, Esophageal varices; GV, Gastric varices; ALT, Alanine transaminase; PLT, Platelet; LMR, Lymphocyte-monocyte ratio; BMI, Body mass index; HE, Hepatic encephalopathy; NLPR, Neutrophil-lymphocyte-platelet ratio; AFP, Alpha-fetoprotein; ALB, Albumin; IgG, Immunoglobulin G; HIV, Human immunodeficiency virus; PSE, Partial splenic embolization; APRI, Aspartate aminotransferase to platelet ratio index; NLR, Neutrophil-lymphocyte ratio; PVD, Portal vein diameter; CHE, Cholinesterase.

**Supplementary Figure 1. Sensitivity analysis of the overall recompensation prevalence in decompensated cirrhosis**

**Supplementary Figure 2. (A) Sensitivity analysis of the recompensation prevalence in Eastern patients with decompensated cirrhosis; (B) Sensitivity analysis of the recompensation prevalence in Western patients with decompensated cirrhosis**

**Supplementary Figure 3. Sensitivity analysis of the recompensation prevalence in patients with decompensated cirrhosis across different etiological subgroups. (A) HBV-related decompensated cirrhosis; (B) HCV-related decompensated cirrhosis; (C) AILD-related decompensated cirrhosis; (D) ALD-related decompensated cirrhosis**

**Supplementary Figure 4. The forest plot summarizes the odds ratios for predictors associated with recompensation in decompensated cirrhosis. (A) Gender; (B) Platelet; (C) Albumin; (D) MELD; (E) CTP; (F)ALT**

**Supplementary figure 5. Sensitivity analysis of prognostic hazard ratio after recompensation in decompensated cirrhosis**
